# Interaction effects of anxiety and outdoor activity spaces on frailty among nursing home residents in Jinan, China: Is there a gender difference?

**DOI:** 10.3389/fpubh.2023.1133340

**Published:** 2023-02-24

**Authors:** Meng Zhao, Tiange Qu, Yang Li, Yaqi Wang, Ming Li, Kefang Wang

**Affiliations:** School of Nursing and Rehabilitation, Cheeloo College of Medicine, Shandong University, Jinan, Shandong, China

**Keywords:** anxiety, outdoor activity spaces, frailty, older adults, gender differences

## Abstract

**Background:**

Anxiety and the physical environment are critical factors influencing frailty among older adults; however, the interaction effect of anxiety and the physical environment, such as outdoor activity spaces, on frailty has not been examined. This study aimed to investigate the interaction effect of anxiety and outdoor activity spaces on frailty and to identify differences by gender.

**Methods:**

A total of 353 nursing home residents (197 women; 156 men; age ≥ 60 years) from 27 Chinese nursing homes were included in the analysis. Anxiety and frailty were analyzed using the Generalized Anxiety Disorder Scale and the FRAIL-NH Scale, respectively. Outdoor activity spaces were assessed through on-site observations using self-designed items. Demographic and socioeconomic information and health-related covariates were also collected. Interaction effect analyses were conducted using multilevel mixed-effects linear models.

**Results:**

Anxiety and outdoor activity spaces had an interaction effect on frailty among nursing home residents (β = −1.32, 95% CI: −2.44, −0.20). However, further analysis demonstrated that this interaction effect was only significant in older women (β = −1.60, 95% CI: −2.93, −0.27) but not in older men (β = −0.23, 95% CI: −2.29, 1.82).

**Conclusions:**

This study highlighted that gender differences should be considered when preventing frailty in older adults with anxiety. Furthermore, it may be beneficial for nursing homes to provide outdoor activity spaces and create a supportive living environment to help delay or reverse frailty among female nursing home residents.

## 1. Introduction

Frailty is a potentially reversible state characterized by declined physiological reserves across multiple systems and accompanied by increased vulnerability to stressors (1). Individuals with frailty have an elevated risk of adverse health outcomes, including falls, hospitalization, disability, and mortality (1, 2). Compared with community-dwelling older adults, individuals residing in nursing homes might be more vulnerable and tend to simultaneously have multiple risk factors for frailty, such as comorbidities and malnutrition. The pooled prevalence of frailty in nursing homes residents is 52.3%, which is much higher than the 10.7% reported in community settings (3, 4). Accordingly, the prevention and management of frailty could be more challenging in nursing homes. Identifying modifiable frailty factors is the first step toward formulating primary prevention and restorative strategies.

Anxiety is strongly associated with accelerated frailty and poorer health status in later life (5, 6). Anxiety has been described as a “silent geriatric giant,” (7) as it is highly prevalent among older adults. Approximately 6.5–58.4% of nursing home residents are reported to experience anxiety (8, 9); however, they rarely seek help for it (10). Older adults with anxiety are more likely to report and experience health problems such as falls, pain, and chronic illnesses (11, 12), which further contribute to the occurrence of frailty. A prospective cohort study in western China including 4,103 community-dwelling older adults demonstrated that individuals with comorbid depressive and anxiety symptoms were seven times more likely to become frail than those without (5). Another study in older surgical patients revealed that anxiety was related to frailty (6). Previous studies have shown that the impact of anxiety on frailty may be reversible and avoidable (13), suggesting that anxiety is potentially a remedial risk factor for frailty. However, few studies have investigated the protective factors that help reduce frailty in older adults with anxiety. As such, it is valuable to consider factors that may aid in buffering the detrimental effect of anxiety on frailty in later life.

In more recent years, contexts have received increasing attention for their role in frailty. An emerging body of research has focused on exploring the association between neighborhood environments and frailty. Specifically, life spaces, walking environments, aesthetic quality, accessible exercise facilities, and basic infrastructure in neighborhoods are important contributors to the level of frailty (14–16). For nursing home residents, the living environment is also recognized as a vital context for residents' health. This is because they typically spend a great deal of time there and rely heavily on social connections and resources to maintain health because of their limited mobility and functioning. Particularly, during the coronavirus disease 2019 pandemic, older adults were restricted from leaving nursing homes, and institutional outdoor spaces were one of the few places for older adults to spend time outside. Hence, high exposure to outdoor environments may potentially affect the health status of nursing home residents. Theories of environmental gerontology state that individuals are influenced by an ongoing interaction between individual, social, and physical environments (17). As a consistent and proximate aspect of the physical environment, outdoor activity spaces may mitigate the effects of anxiety on frailty.

Furthermore, no studies have assessed whether the interaction effect of anxiety and outdoor activity spaces on frailty may vary by gender. Numerous studies have revealed significant gender differences in anxiety and frailty, with women having higher odds of being anxious and frail than men (18–20). Significant gender differences were also found in the association between anxiety and frailty. Compared with older men without comorbid depression and anxiety, women with anxiety alone had a higher prevalence of frailty (11). Previous literature has reported gender differences in the effect of the neighborhood environment on health. For instance, Stafford et al. (21) showed that physical characteristics of the neighborhood were more strongly associated with women's than men's health. They suggest that the residential environment may be more important for women's health, perhaps because women have greater exposure to their neighborhood environment or are more vulnerable to its effects. Given these findings, we speculate that the interaction effect of outdoor activity spaces and anxiety on frailty is greater for women than men.

Therefore, we hypothesized that outdoor activity spaces play a role in buffering against the adverse effects of anxiety on frailty, which may exert a stronger buffering role in older women than in male residents. This study aimed to explore the interaction effect of outdoor activity spaces and anxiety on frailty among nursing home residents in China and analyse potential gender differences.

## 2. Materials and methods

### 2.1. Participants

This cross-sectional, descriptive study was conducted among nursing homes in five districts (Lixia, Tianqiao, Huaiyin, Shizhong, and Licheng) in Jinan, Shandong Province, China, from March to June 2018. Twenty-seven nursing homes were sampled for the study from the 69 nursing homes registered at the Civil Affairs Bureau with more than 30 beds and that have operated for longer than a year. Forty-two were excluded for the following reasons: refusing to participate (*n* = 28), relocating or renovating (*n* = 6), and having missing contact details (*n* = 8).

**Figure 1:**
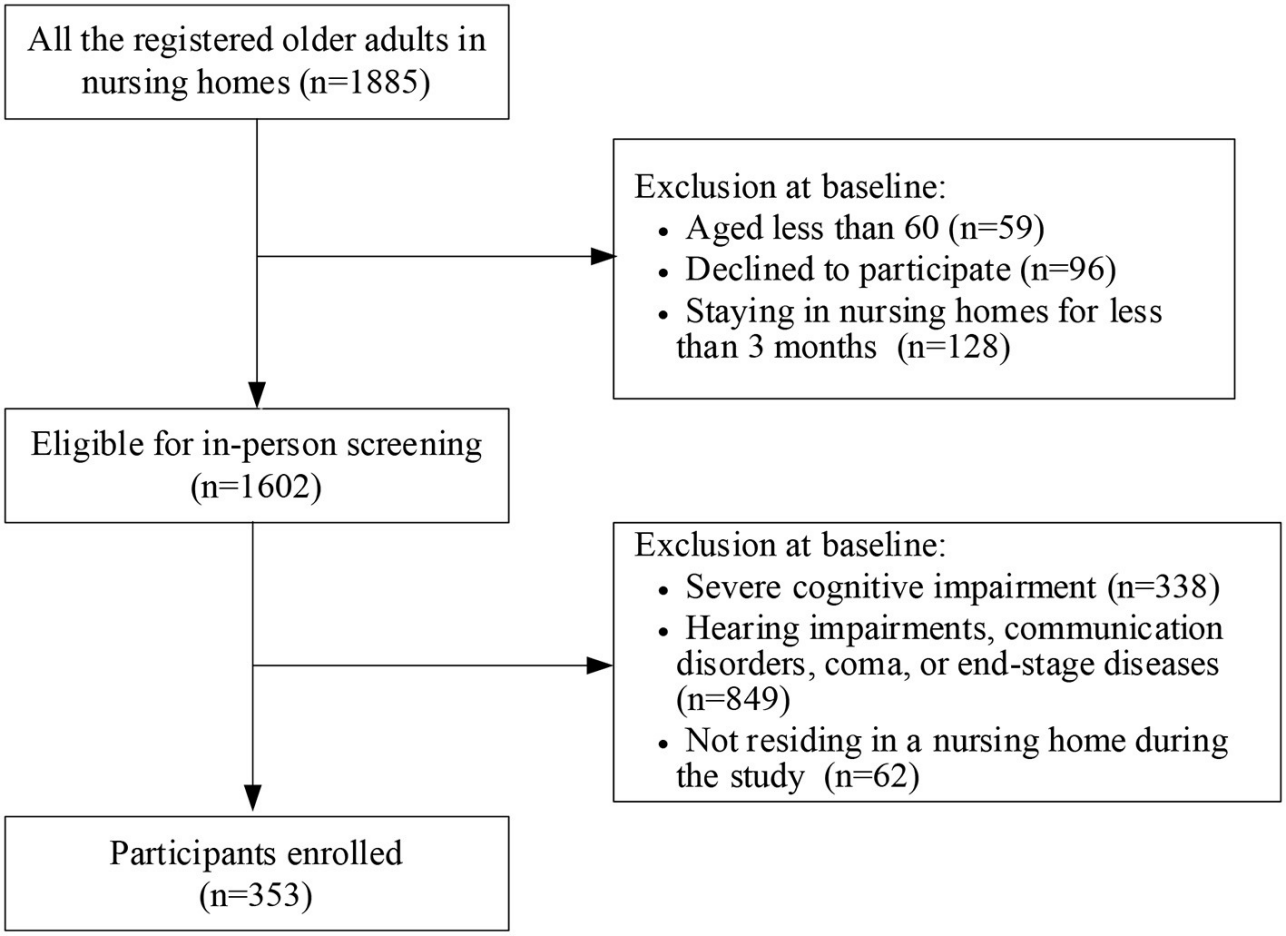
Flowchart depicting participant selection process.

Only residents aged ≥60 years who had been residing in a nursing home for ≥3 months were included in this study. Exclusion criteria were (i) hearing impairments, communication disorders, comatose, or end-stage diseases; (ii) not residing in a nursing home during the study; and (iii) severe cognitive dysfunction as determined by a Mini-Mental State Examination (MMSE) score <10 (22). In total, 353 eligible residents were invited to participate. Details on the participant enrolment process are shown in Figure 1.

All participants were fully informed regarding the study and provided written informed consent. The study was approved by the Ethics Committee of the researchers' university (approval number 2017-R-112).

### 2.2. Data collection

The collection was collected anonymously. Prior to the survey, research assistants (well-trained nursing postgraduates and undergraduates) received uniform training on conducting structured face-to-face interviews and physical performance measurements, and they were asked to follow a standardized questioning sequence. After passing a minimum of 6 h of training, the research assistants were allowed to conduct the survey independently.

### 2.3. Measures

#### 2.3.1. Exposure of interest: Anxiety

Anxiety was measured using the two-item Generalized Anxiety Disorder Scale (23). Participants were asked how frequently symptoms of anxiety bothered them over the past 2 weeks (0 = “not at all;” 1 = “several days;” 2 = “more than half the days;” 3 = “nearly every day.” A total score of 0 to 2 was defined as “no anxiety,” whereas a score of 3–6 was defined as “anxiety”).

#### 2.3.2. Outcome of interest: Frailty

Frailty was defined using the Chinese version of the FRAIL-NH scale (24). The FRAIL-NH scale, which includes core elements of the frailty phenotype and frailty index, is a specific measurement tool for nursing home residents. It comprises seven components: fatigue, resistance, ambulation, incontinence, weight loss, nutritional approach, and help with dressing. Each component is graded as 0, 1, or 2. The total score ranges from 0 to 14, with higher scores indicating a higher likelihood of frailty.

#### 2.3.3. Moderator of interest: Outdoor activity spaces

Outdoor activity spaces were investigated through on-site observations by research assistants. Nursing homes were considered to provide outdoor activity spaces if they contained basic and durable fitness amenities or recreational facilities, usually installed in open spaces, such as outdoor courtyards, including spacewalk machines, leg presses, treadmills, and rotary torso machines.

#### 2.3.4. Covariates

A priori, we identified potential covariates for adjustment based on the knowledge of factors that might causally affect the study exposure and study outcome independent of the exposure. The demographic and socioeconomic covariates were age (years), years of education (years), marital status (married vs. single/divorced /widowed), and self-reported economic conditions (good vs. poor).

The health-related covariates included comorbidities, cognitive impairment, loneliness, and nutritional status. Comorbidities were defined as the presence of two or more chronic diseases (25). Cognitive status was assessed using the MMSE, with scores <24 indicating cognitive impairment (22). Loneliness was measured using a common five-point Likert scale that asked residents how often they felt lonely (26). This variable was dichotomised prior to statistical analyses: “sometimes,” “often,” and “always” represented loneliness, whereas “seldom” or “never” represented no loneliness. Nutritional status was determined using the Mini Nutritional Assessment-Short Form (27). The total scores for this assessment ranged from 0 to 14, with higher scores denoting better nutritional status.

### 2.4. Statistical analyses

Participant characteristics were presented as means (standard deviations) for continuous variables and frequencies (percentages) for categorical variables. Independent sample *t*-tests for continuous variables and the chi-squared or Fisher's exact tests for categorical variables were used to examine the differences in characteristics between men and women. As residents were clustered within nursing homes, multilevel mixed-effects linear regression models were constructed based on three models to test the proposed hypotheses. Model 1 was unadjusted, Model 2 included sociodemographic covariates, and Model 3 was additionally adjusted for health-related covariates. Furthermore, a margin plot was utilized to illustrate the interaction effect of anxiety and outdoor activity spaces. All analyses were performed using stratified analyses of gender to allow for possible differences in the subgroups.

We performed sensitivity analysis to evaluate the consistency of the results. To reduce the potential for reverse causality between anxiety and frailty, multilevel mixed-effects logistic regression models were re-estimated to identify the potential interaction effect between frailty and outdoor activity spaces on anxiety as well as gender differences.

Analyses were performed using Stata, version 14.1 (Stata Corp, College Station, TX). All statistical tests were two-sided, and *p* < 0.05 was considered significant.

## 3. Results

Table 1 shows the descriptive characteristics of the study population. The age range of the participants was 60–103 years, with a mean age of 79.01 years, and 55.81% were women. Fifty-five participants were unable to ambulate. In total, 19.29% of women and 14.10% of men participants reported anxiety. Of the 27 nursing homes, only 15 had outdoor activity spaces. The mean frailty scores were ~2.37 and 2.26 for women and men, respectively. Women were generally more likely to be older and single than men. Moreover, women had lower education levels, better economic conditions, worse cognitive impairment, less loneliness, and worse nutritional status than men.

**Table 1:** Participant characteristics.

**Variables**	**Overall (*n* = 353)**	**Female (*n* = 197)**	**Male (*n* = 156)**	***p***
	**Mean (*****SD*****) or** ***n*** **(%)**	**Mean (*****SD*****) or** ***n*** **(%)**	**Mean (*****SD*****) or** ***n*** **(%)**	
Age (years)	79.01 (8.80)	80.93 (7.82)	76.58 (9.38)	< 0.001
Years of education	5.26 (4.84)	4.52 (4.52)	8.47 (4.33)	<0.001
Marital status				0.001
Married	63 (17.8)	23 (11.68)	40 (25.64)	
Single/divorced/widowed	290 (82.2)	174 (88.32)	116 (74.36)	
Economic conditions				0.049
Good	131 (37.1)	82 (41.62)	49 (31.41)	
Poor	222 (62.9)	115 (58.38)	107 (68.59)	
Comorbidities				0.078
Yes	265 (75.1)	155 (78.68)	110 (70.51)	
No	88 (24.9)	42 (21.32)	46 (29.49)	
Cognitive impairment^*^				<0.001
Yes	212 (60.2)	95 (48.73)	45 (28.85)	
No	140 (39.8)	101 (51.27)	111 (71.15)	
Loneliness^*^				0.009
Yes	103 (29.4)	46 (24.87)	57 (36.54)	
No	247 (70.6)	148 (75.13)	99 (63.46)	
Nutritional status	9.35 (2.19)	8.99 (2.28)	9.79 (2.01)	0.001
Anxiety				0.254
Yes	60 (17.0)	38 (19.29)	22 (14.10)	
No	293 (83.0)	159 (80.71)	134 (85.90)	
Outdoor activity spaces				0.255
Provided	233 (66.0)	125 (63.45)	108 (69.23)	
None	120 (34.0)	72 (36.55)	48 (30.77)	
Frailty^*^	2.32 (2.49)	2.37 (2.54)	2.26 (2.43)	0.695

SD, standard deviation.

* Missing values (1 for cognitive impairment, 3 for loneliness and frailty).

The results for the association between anxiety and frailty are presented in Table 2. In the overall sample, individuals with anxiety had an increased risk of frailty compared with participants without anxiety in both unadjusted and adjusted models. Further stratification of the association according to gender revealed that men and women who experienced anxiety were at greater risk of frailty in Models 1 and 2 (women: β = 1.91/1.92, 95% CI: 1.07/1.09, 2.75/2.75; men: β = 1.65/1.50, 95% CI: 0.59/0.44, 2.71/2.57). After adjusting for all identified sociodemographic and health-related covariates, women who reported anxiety had a significantly higher risk of frailty (β = 1.25, 95% CI: 0.59, 1.90), whereas men did not (β = 0.76, 95% CI: −0.12, 1.63).

**Table 2:** Association between anxiety and frailty.

**Model**	**Overall (*n* = 353)**	**Female (*n* = 197)**	**Male (*n* = 156)**
	β **(95% CI)**	β **(95% CI)**	β **(95% CI)**
**Model 1**			
**Anxiety (ref. no)**			
Yes	1.86 (1.20, 2.52)^‡^	1.91 (1.07, 2.75)^‡^	1.65 (0.59, 2.71)^†^
**Model 2**			
**Anxiety (ref. no)**			
Yes	1.80 (1.14, 2.46)^‡^	1.92 (1.09, 2.75)^‡^	1.50 (0.44, 2.57)^†^
**Model 3**			
**Anxiety (ref. no)**			
Yes	1.09 (0.56, 1.62)^‡^	1.25 (0.59, 1.90)^‡^	0.76 (-0.12, 1.63)

Model 1 was unadjusted; Model 2 was adjusted for sociodemographic covariates (age, years of education, marital status, and economic conditions); Model 3 was further adjusted for health-related covariates (comorbidities, cognitive impairment, loneliness, and nutritional status).

β, regression coefficient; CI, confidence interval; ref, reference group.

† p < 0.01,

‡ p < 0.001.

**Figure 2:**
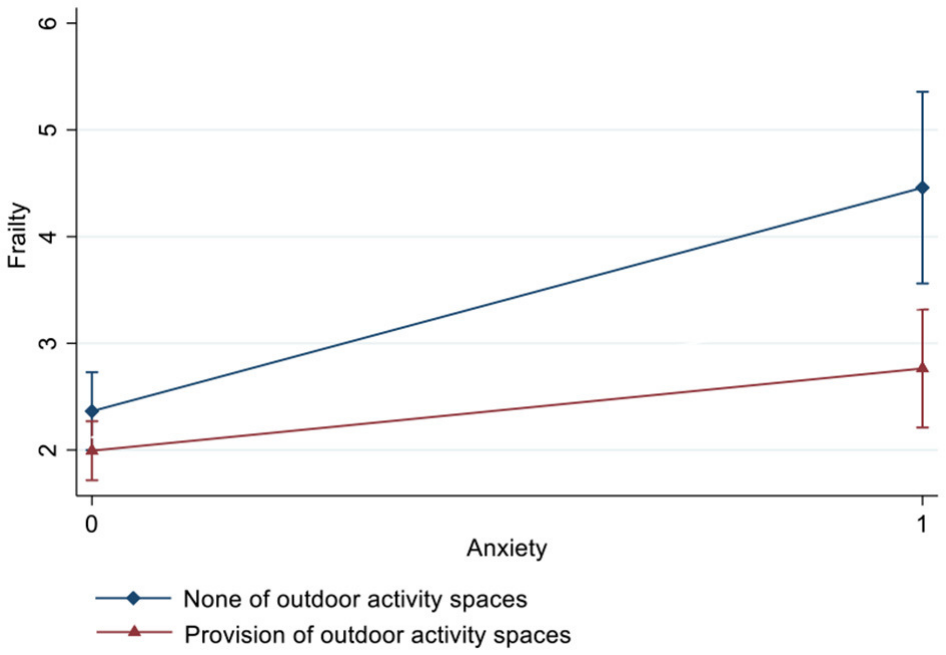
Interaction effect of anxiety and outdoor activity spaces on frailty estimated based on a fully adjusted model (including age, years of education, marital status, economic conditions, comorbidities, cognitive impairment, loneliness, and nutritional status).

**Figure 3:**
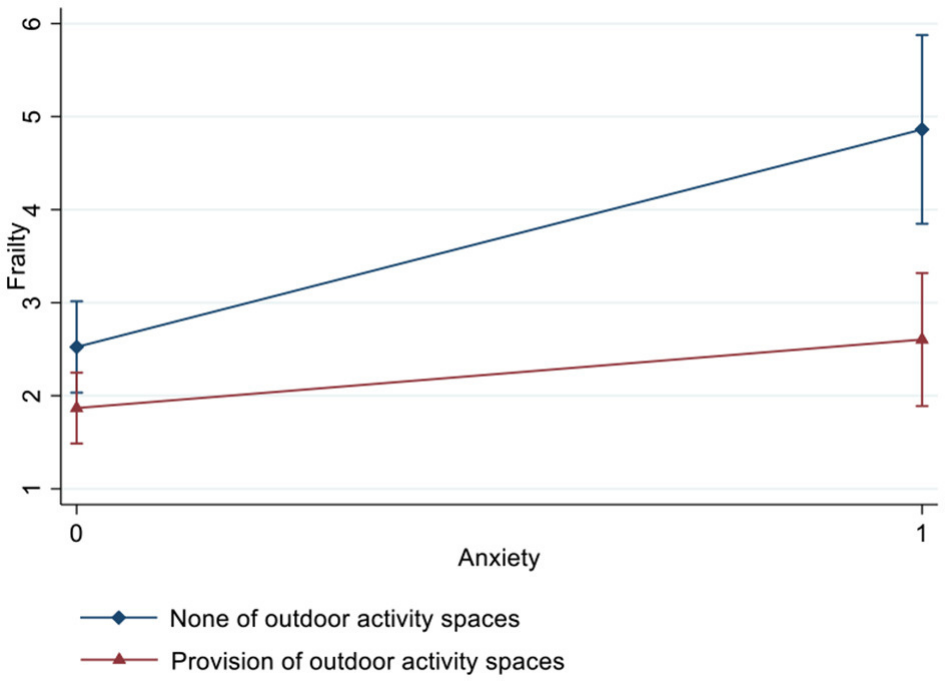
Interaction effect of anxiety and outdoor activity spaces on frailty among women estimated based on a fully adjusted model (including age, years of education, marital status, economic conditions, comorbidities, cognitive impairment, loneliness, and nutritional status).

**Figure 4:**
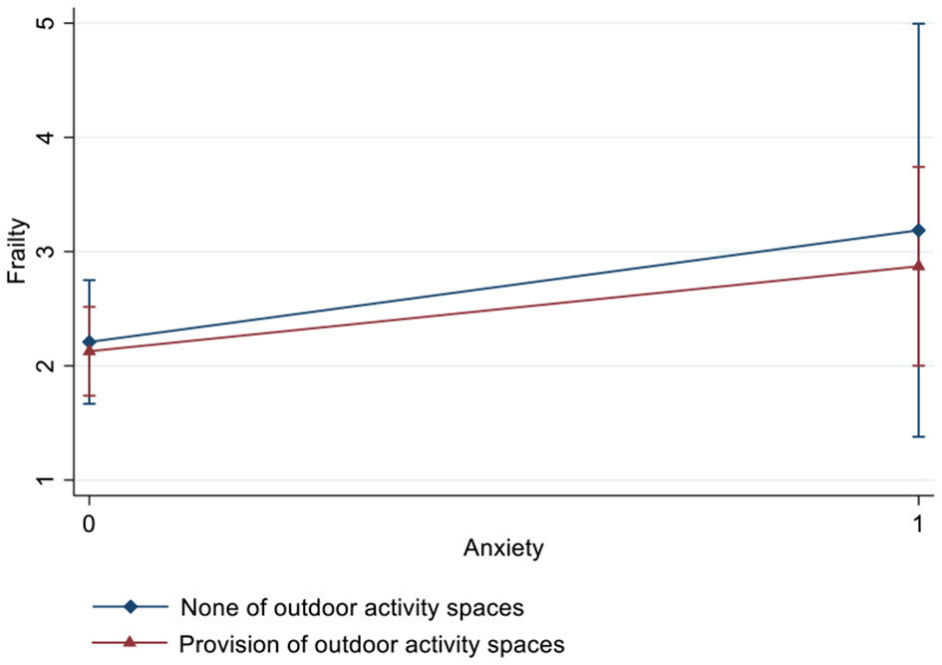
Interaction effect of anxiety and outdoor activity spaces on frailty among men estimated based on a fully adjusted model (including age, years of education, marital status, economic conditions, comorbidities, cognitive impairment, loneliness, and nutritional status).

Table 3 and Figures 2–4 show the interaction effects of anxiety and outdoor activity spaces on frailty as well as gender differences. For the overall sample and for women, the significant interaction term suggested that outdoor activity spaces played a moderating role in anxiety and frailty (β = −1.32/−1.60, 95% CI: −2.44/−2.93, −0.20/−0.27). For instance, if nursing homes provided outdoor activity spaces, women with anxiety were less likely to develop frailty. However, among men, there was no significant interaction effect between anxiety and outdoor activity spaces (β = −0.23, 95% CI: −2.29, 1.82).

**Table 3:** Interaction effect of anxiety and outdoor activity spaces on frailty.

**Model**	**Overall (*n* = 353)**	**Female (*n* = 197)**	**Male (*n* = 156)**
	β **(95% CI)**	β **(95% CI)**	β **(95% CI)**
**Model 1**			
**Anxiety (ref. no)**			
Yes	2.94 (1.77, 4.11)^‡^	3.35 (2.00, 4.69)^‡^	1.57 (−0.79, 3.93)
**Outdoor activity spaces (ref. no)**			
Provided	−1.19 (−1.76, −0.63)^‡^	−1.45 (−2.26, −0.64)^‡^	−0.97 (−1.81, −0.14)^*^
Anxiety × outdoor activity spaces	−1.42 (−2.82, −0.03)^*^	−2.09 (−3.76, −0.43)^*^	0.28 (−2.35, 2.91)
**Model 2**			
**Anxiety (ref. no)**			
Yes	2.86 (1.71, 4.01)^‡^	3.25 (1.95, 4.55)^‡^	1.40 (−0.98, 3.77)
**Outdoor activity spaces (ref. no)**			
Provided	−1.12 (−1.66, −0.58)^‡^	−1.37 (−2.12, −0.62)^‡^	−0.93 (−1.76, −0.10)^*^
Anxiety × outdoor activity spaces	−1.43 (−2.79, −0.06)^*^	−2.08 (−3.71, −0.45)^*^	0.33 (−2.28, 2.94)
**Model 3**			
**Anxiety (ref. no)**			
Yes	2.09 (1.14, 3.05)^‡^	2.34 (1.25, 3.42)^‡^	0.98 (−0.90, 2.85)
**Outdoor activity spaces (ref. no)**			
Provided	−0.37 (−0.84, 0.10)	−0.66 (−1.29, −0.03)^*^	−0.08 (−0.77, 0.60)
Anxiety × outdoor activity spaces	−1.32 (−2.44, −0.20)^*^	−1.60 (−2.93, −0.27)^*^	−0.23 (−2.29, 1.82)

Model 1 was unadjusted; Model 2 was adjusted for sociodemographic covariates (age, years of education, marital status, and economic conditions); Model 3 was further adjusted for health-related covariates (comorbidities, cognitive impairment, loneliness, and nutritional status).

β, regression coefficient; CI, confidence interval; ref, reference group.

* p < 0.05,

‡ p < 0.001.

The sensitivity analysis revealed no interaction effect between frailty and outdoor activity spaces on anxiety and no gender differences (Supplementary Table 1).

## 4. Discussion

With an aging population in many countries, frailty has increasingly become an emerging public health issue. Environmental and individual factors are fundamental causes of frailty (2, 16). To the best of our knowledge, only a few studies to date have explored the cross-level interaction effects of individual factors and environmental elements on frailty as well as corresponding gender differences. This study investigated the interaction effect of anxiety and outdoor activity spaces on frailty from the perspective of individual and environment interactions and examined potential gender differences. The results demonstrated that the interaction between anxiety and outdoor activity spaces was a significant predictor of frailty. However, the interaction effect was only observed among older women with anxiety and not older men with anxiety. This study provides practical guidance that may help nursing homes take measures to prevent and mitigate frailty among residents with anxiety. We hope that this study will encourage more researchers to explore frailty from the perspective of the interaction between the individual and the environment.

Consistent with previous findings (11, 12), our results demonstrated that individuals with anxiety were more likely to develop frailty. Although the exact mechanisms remain unclear, there are several possible underlying pathophysiological mechanisms. Growing evidence supports a positive association between anxiety and inflammatory cytokines, such as interleukin-6 and C-reactive protein (28, 29), which are known to be elevated in individuals with frailty (2, 30). Another explanation is hypothalamic-pituitary-adrenal (HPA) axis dysregulation. A study with older adults in Spain suggested that serum cortisol concentration was related to increasing frailty burden (31). Another study with residents of long-stay institutions in Brazil reported that salivary cortisol levels were positively associated with frailty (32). In addition, a population-based study in the Netherlands reported that older adults with anxiety had a lower cortisol awakening response than those without (33). These findings suggest that HPA axis dysregulation may increase the vulnerability of older adults to anxiety and frailty. However, in the fully adjusted model, which considered gender differences, the association between anxiety and frailty was significant only among women. Similarly, several extant studies indicated that women with mental disorders had higher levels of frailty than men (11, 34). One possible explanation for gender differences is that women in China have lower incomes and education levels and are more likely to be widowed than men (35, 36). These factors could contribute to anxiety. Furthermore, psychological distress may increase the likelihood of frailty in women. Another possible explanation primarily linked to gender differences is biological susceptibility. Compared with men, women with anxiety reported higher serum high-sensitivity C-reactive protein (29) and diurnal cortisol levels (37), leading to loss of muscle mass, muscle strength, weight loss, and reduced energy expenditure (30), all of which are key clinical features of frailty.

We observed a significant buffering effect of outdoor activity spaces on the association between anxiety and frailty, which is supported by the aforementioned theories of environmental gerontology (17). Outdoor activity spaces provide an incentive for being physically active (38, 39), and serve as places for individuals to walk, run, dance, and perform other activities. This, in turn, may contribute to reducing anxiety and frailty risks by promoting a healthy lifestyle. Further, stress reduction theory indicates that exposure to outdoor environments may trigger the parasympathetic nervous system to reduce negative mental health outcomes, such as stress and anxiety (40). Studies have reported that access to outdoor environments is psychologically restorative and promotes mental health (39). Moreover, outdoor activity spaces create a platform for older adults to communicate and interact with others. Through the outdoor activity spaces provided by nursing homes, older adults can get out of their small living spaces and engage in social contact, which can reduce the anxiety elicited by the new environment and delay frailty. Thus, outdoor activity spaces could reduce the likelihood of frailty among older adults with anxiety.

Gender-stratified analysis indicated that outdoor activity spaces seemed to only reduce the harmful effects of anxiety on frailty in women. Self-construal theory contains an important factor that may account for this finding (41, 42). According to this theory (41, 42), men are more likely to develop and maintain an independent self-construal, in which others are represented as separate from the self, whereas women tend to develop and maintain an interdependent self-construal, in which others are represented as part of the self (41, 42). These gender differences in self-construal could lead to divergent coping behaviors in response to psychological symptoms. Specifically, when men experience anxiety, they are more likely to actively self-regulate and cope independently and assertively. Conversely, women with anxiety are sensitive and tend to seek emotional support and social connections from interaction with others. Strong evidence exists that frailty can be prevented by an increase in social contacts (2, 19), which is more likely to occur with outdoor activity spaces, as they can facilitate social interaction. In addition, although we did not collect any information regarding the usage of outdoor activity spaces, our supplementary analysis revealed that women had a higher level of physical activity and were more likely than men to have spent their leisure time outdoors (data not shown); therefore, women could benefit more from outdoor activity spaces than men. Moreover, a recent study demonstrated that female nursing home residents had significantly higher engagement in physical activity than men (43). Physical activity is considered a promising method to reverse frailty (2); hence, outdoor activity spaces are more likely to buffer the risk of frailty in older women with anxiety.

Several limitations of this study should be acknowledged when interpreting the findings. First, the data were collected at a single time point, providing useful information about their associations but precluding any assertions of causality. Further longitudinal studies are required to establish causality by using measures at various time points. Second, participants were selected from one relatively economically developed city in China, which limits the generalisability of the study findings. Future studies are required to replicate these findings with a larger, more diverse sample of older adults, considering the great diversity in the levels of economic development, such as between urban and rural areas. Third, although we collected information on outdoor activity spaces, data on various facilities, hygiene practices, and aesthetic features of outdoor activity spaces were not available for analysis, which may affect the association between anxiety and frailty. These factors merit attention in future studies.

Despite these limitations, this study provides a novel perspective for improving the wellbeing of older adults and promoting healthy aging. Providing outdoor activity spaces could be an effective mechanism to prevent frailty among older women with anxiety. We suggest that policymakers and local governments supervise and guide nursing homes to equip outdoor activity spaces and implement activity plans, as our results found that only 15 of the 27 surveyed nursing homes provided outdoor activity spaces. In addition, some nursing homes had a small area of per capita activity space, and some were reconstructed from old buildings without consideration for outdoor activity spaces, which seriously limited residents' activities. Outdoor activity spaces could also be considered as indicators for nursing home quality evaluation. Moreover, we suggest that health providers and nursing home staff encourage older women with anxiety to visit and relax in outdoor activity spaces, as nursing home residents spend up to 65% of their time alone and are often physically inactive in their rooms (44).

In conclusion, this study represents an important first step in providing generalizable evidence regarding the effect of outdoor activity spaces on the relationship between anxiety and frailty among older men and women. Our findings highlighted the buffering effect of outdoor activity spaces on frailty among older women with anxiety but not among men. Moreover, the study results suggest that gender differences should be considered in the prevention of frailty in older adults with anxiety. Furthermore, it may be beneficial for nursing homes to provide outdoor activity spaces and create a supportive living environment to delay or reverse frailty among female nursing home residents.

## Data availability statement

The raw data supporting the conclusions of this article will be made available by the authors, without undue reservation.

## Ethics statement

The study was approved by the Ethics Committee of the Shandong University (approval number 2017-R-112). The patients/participants provided their written informed consent to participate in this study.

## Author contributions

ML and KW: supervision, writing-reviewing and editing, and critical revision. MZ: funding acquisition, conceptualization, methodology, investigation, data curation, writing-original draft preparation, and writing-reviewing and editing. TQ: investigation and methodology. YL: investigation. YW: conceptualization, investigation, and methodology. All authors contributed to the article and approved the submitted version.
