# Supplementary material for: Interaction effects of anxiety and outdoor activity spaces on frailty among nursing home residents in Jinan, China: Is there a gender difference?
Source: Front Public Health. 2023 Feb 24;11:1133340. doi: 10.3389/fpubh.2023.1133340 (PMC9999001; doi:10.3389/fpubh.2023.1133340)
Supplement: Supplementary file 1 [file Table_1.docx]

Supplementary Material

**Supplementary Table 1**. Interaction effect of frailty and outdoor activity spaces on anxiety

| Model | Overall (n = 353) |  | Female (n = 197) |  | Male (n = 156) | |
| --- | --- | --- | --- | --- | --- | --- |
|  | β (95% CI) |  | β (95% CI) |  | β (95% CI) | |
| **Model 1** |  |  |  |  |  |  |
| Frailty (ref. no) | |  |  |  |  |  |
| Yes | 0.04 (0.02, 0.07)‡ |  | 0.06 (0.03, 0.09)‡ |  | 0.02 (-0.02, 0.05) | |
| Outdoor activity spaces (ref. no) | |  |  |  |  |  |
| Provided | 0.10 (-0.03, 0.23) |  | 0.15 (-0.02, 0.31) |  | 0.02 (-0.14, 0.18) | |
| Frailty × outdoor activity spaces | 0.01 (-0.03, 0.04) |  | -0.01 (-0.06, 0.03) |  | 0.04 (-0.01, 0.08) | |
| **Model 2** |  |  |  |  |  |  |
| Frailty (ref. no) |  |  |  |  |  |  |
| Yes | 0.04 (0.02, 0.06)‡ |  | 0.06 (0.03, 0.09)‡ |  | 0.01 (-0.02, 0.05) | |
| Outdoor activity spaces (ref. no) | |  |  |  |  |  |
| Provided | 0.08 (-0.03, 0.21) |  | 0.12 (-0.04, 0.29) |  | 0.03 (-0.13, 0.19) | |
| Frailty × outdoor activity spaces | 0.01 (-0.03, 0.04) |  | -0.01 (-0.06, 0.03) |  | 0.04 (-0.01, 0.08) | |
| **Model 3** |  |  |  |  |  |  |
| Frailty (ref. no) |  |  |  |  |  |  |
| Yes | 0.04 (0.01, 0.64)† |  | 0.06 (0.02, 0.09)† |  | 0.01 (-0.03, 0.04) | |
| Outdoor activity spaces (ref. no) | |  |  |  |  |  |
| Provided | 0.09 (-0.04, 0.22) |  | 0.10 (-0.07, 0.27) |  | 0.08 (-0.07, 0.23) | |
| Frailty × outdoor activity spaces | 0.01 (-0.02, 0.04) |  | -0.01 (-0.05, 0.04) |  | 0.03 (-0.01, 0.07) | |

Model 1 was unadjusted; Model 2 was adjusted for sociodemographic covariates (age, years of education, marital status, and economic conditions); Model 3 was further adjusted for health-related covariates (comorbidities, cognitive impairment, loneliness, and nutritional status).

β = regression coefficient; CI = confidence interval; ref = reference group.

**p*﹤.05, †*p*﹤.01, ‡*p*﹤.001
